# REACh for the preschoolers; a developmental assessment tool for 2–5 year old children in Sri Lanka

**DOI:** 10.1186/s12887-023-03895-5

**Published:** 2023-02-16

**Authors:** A.V Caldera, A. R Wickremasinghe, N Muttiah, P. K. S Godamunne, B.N Jayasena, L. K. E Chathurika, K. M. N Perera, M Mendis, D Tilakarathne, M. K. R.R Peiris, T Wijesinghe, N.E Senarathna, W. D. L Saubhagya, M Chandraratne, S.P Sumanasena

**Affiliations:** 1 Department of Public Health, Faculty of Medicine, University of Kelaniya, Ragama, Sri Lanka; 2 Department of Disability Studies, Faculty of Medicine, University of Kelaniya, Ragama, Sri Lanka; 3 Department of Medical Education, Faculty of Medicine, University of Kelaniya, Ragama, Sri Lanka; 4 Formerly at PLAN Sri Lanka, Colombo, Sri Lanka; 5 Ministry of Women and Child Affairs, Children’s Secretariat, Colombo, Sri Lanka

**Keywords:** Early Childhood Development, Developmental assessment, Preschool teachers, Developmental Screening, Lower Middle Income Country

## Abstract

**Background:**

Preschool children in low resource settings are at higher risk of missing developmental potential due to the lack of standardized and validated methods for the timely detection of children with developmental delays or neurodevelopmental disorders. The preschool teacher is a non-specialist resourceful link within the community to detect and offer interventions early. This paper discusses the preliminary iteration of designing and testing the psychometric properties of a developmental assessment for children aged 24 to 60 months in Sri Lanka. This assessment is designed to be conducted by preschool teachers in their preschool setting.

**Methods:**

Three processes followed: 1. Designing and development of the Ragama Early Assessment for Children (REACh) complete preschool developmental assessment and a tool kit 2. Testing and training teachers on conducting the REACh assessment 3. Preliminary assessment of the psychometric properties including content validity, internal consistency, interrater reliability and concurrent validity.

**Results:**

A literature search identified 11 assessments and 542 items representing cognitive, social-emotional and adaptive, language and motor domains. Content validity was assessed to select and adapt items. A complete assessment tool was designed to be administered in four settings within the preschool. This was further improved during pre and pilot testing and teacher training. Cronbach's alpha measuring internal consistency was > 0.70 for cognitive, language, social-emotional and adaptive domains across all three age groups in 1809 children. Interrater reliability was > 65% for age groups 36–47 and 47- 60 months. Concurrent validity using a clinical gold standard demonstrated sensitivity of more than 0.75 for all age groups with variable specificities (24–35 months: 0.71, 36- 47 months: 0.43 and 48–60 months: 0.67) assessed in 75 children.

**Conclusions:**

This culturally and linguistically adapted tool was tested nationally in Sri Lanka. The inte-rrater reliability between teachers and research assistants was higher than 65% for all domains in children more than 36 months. The preliminary iteration confirms it as an acceptable screening assessment for all age groups but with significantly lower specificity in the 36-47 month age group. Further improvement in certain domains together with intense teacher training is likely to enhance the validity and reliability of the assessment.

**Trial registration:**

Ethics clearance for the procedure was granted prospectively from the Ethics Review Committee, Faculty of Medicine, University of Kelaniya (ERC no. P 131/06/2018).

**Supplementary Information:**

The online version contains supplementary material available at 10.1186/s12887-023-03895-5.

## Background

Approximately, 43% of children under the age of 5 years living in Low Income (LIC) and Lower Middle Income Countries (LMICs) are at higher risk of not reaching their developmental potential [1, 2]. Sri Lanka is a LMIC, with impressive neonatal and infant mortality indices in comparison to many other countries in the region [3]. Reduction in mortality due to better healthcare and advanced technologies have led to survival of infants at risk for neuro-developmental disorders (NDDs) such as cerebral palsy, autism spectrum disorder, learning disorders, and subtle behavior abnormalities [4]. Delays in early detection of such children in LMIC settings may result in NDDs leading to deterioration in health, nutrition, and lack of opportunities for learning and employment [5]. Based on the neural plasticity theory, the brain has an immense potential to rewire and repair from 0–5 years. Hence, early detection and investment on early intervention are critical in providing the best opportunities for activity and participation of children with or at risk of developmental delay and disabilities [5].

Yet, the majority of such settings have numerous challenges in implementing much needed services for these children. Developmental screening and assessments are resource intensive in terms of time and skills, posing multiple difficulties to the non-specialist community workers at the field level [6]. Even in settings with qualified staff, assessments developed for Western countries are often used. These may contain items that are culturally inappropriate and unfamiliar to children in LMICs, and may lack precision, leading to erroneous reporting [6].

Such deficiencies in assessments inevitably lead to delayed detection and under estimation of prevalence and incidence of NDDs in LMICs, especially when features are subtle [7]. Further, such delays will prevent access to specific intervention programmes leading to significant long term social and economic impacts [6, 8].

Given this background, the preschool becomes the ideal context for early identification of children with developmental delays who were undetected during regular health services assessments. Evidence shows positive outcomes through quality preschool education from vulnerable households [9, 10].

The preschool teachers are non-specialist community-based service providers with background skills and knowledge in child development. Therefore, capacity building to use scientific developmental assessments is a possibility. They are capable of making reliable and consistent assessments over a longer period as children spend a considerable amount of time within the preschool setting. The early identification of disabilities in preschools will enable these institutions to deliver more specific child directed early education strategies and better school readiness opportunities enhancing the chances of obtaining inclusive formal education [11]. Such initiatives will contribute towards achieving Sustainable Development Goal 4 (SDG) which aims to provide access to “quality early childhood development, care, and preprimary education” for all children by the year 2030 [12].

In the interest of addressing this need, three institutions in Sri Lanka; the Faculty of Medicine, University of Kelaniya, PLAN Sri Lanka, and the Ministry of Women and Child Affairs came together to design a complete preschool developmental assessment tool kit; REACh (Ragama Early Assessment of Children) assessment. The primary aim of this assessment was to enable preschool teachers to conduct developmental screening assessments in 24–60 months old children to identify children with developmental delays or deviations. This tool will be used as a screening assessment to refer children with concerns to the primary healthcare system. The content validity, internal consistency, inter-rater reliability and concurrent validity were measured to obtain the preliminary validity and reliability.

This manuscript outlines the methodology of the preliminary iteration of the development of the REACh assessment and its psychometric properties. Prior to the development of this assessment, the team of researchers and collaborators identified key strategies that underpinned the principles of this project from conceptualization to development. Additional file 1 indicates the key principles and strategies used in the conceptualization of the REACh assessment [See Additional file 1].

## Methods

**Fig. 1:**
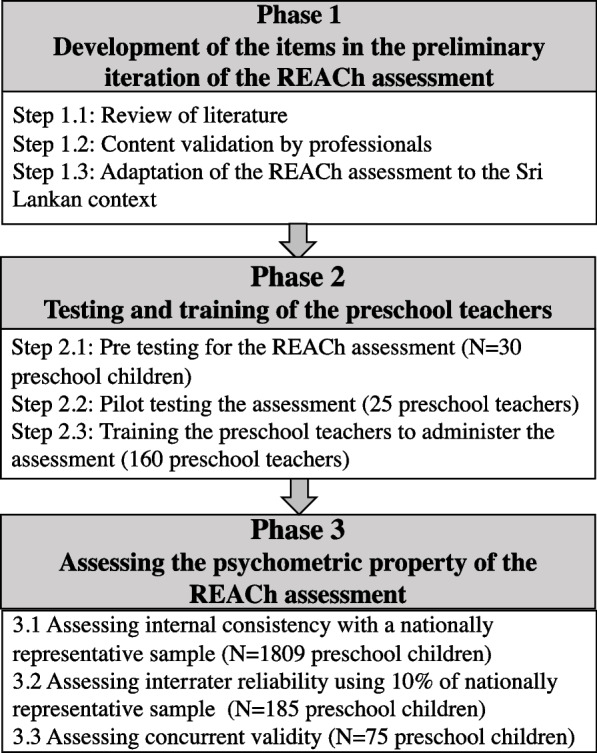
Flow chart representing the phases of the study

The preliminary iteration of the REACh assessment was carried out in 3 phases (Fig. 1), following the assessment development process outlined in literature [13]. The assessment was primarily designed by an academic team following a series of discussions and workshops held with multiple stakeholders. Data collection was carried out by the preschool teachers during phase 3. The members of the research team including trained research assistants (RAs) with a minimum undergraduate qualification, who were speech and language therapists, audiologists and clinical psychologists collected data in phase 3 to establish inter-rater reliability and concurrent validity.

### Phase 1 – Development of items in the preliminary iteration of the REACh assessment

#### Step 1.1- Review of literature

A literature review was conducted on child development assessments published in English for children aged 24–60 months using Google Scholar, Social Science Research Network, PubMed and Web of Science data bases from 2000–2018.

#### Step 1.2- Content validation by professionals

Items selected through the literature review were extensively reviewed through a Delphi process. Each item was reviewed and scored by a group of experts including a developmental pediatrician, a child developmental psychologist, two speech and language pathologists, and two audiologists on a 10-point Likert scale for cultural relevance and clinical importance. Items with an average score less than 7 were eliminated.

A first draft of the assessment was presented to local stakeholders including early childhood officers, preschool teachers, educationists, and health specialists and suggestions were incorporated prior to piloting the assessment. As the primary aim of the project was to develop a national assessment to be used by preschool teachers, the selected items were initially mapped with domains defined within the National Preschool Standards Framework [See Additional file 2].

#### Step 1.3- Adaptation of assessment kit to Sri Lankan context

##### Domains and subsets

The REACh tool consisted of items that were administered by the teachers as well as items scored through classroom observations. Cognitive, language (expressive and receptive), and motor (fine and gross) were always administered items with the exception of a few items from the language domain that required observations. Social-emotional and adaptive skills were primarily assessed through classroom observations with several items administered during the story book reading activity.

##### Assessment settings

**Fig. 2:**
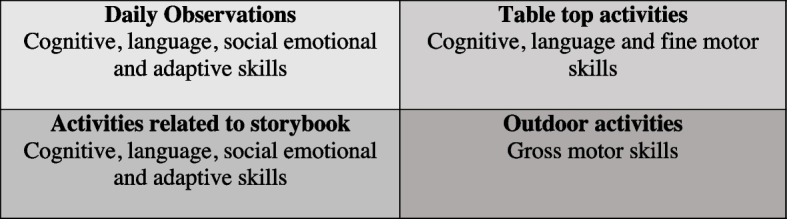
The four settings with the main domains assessed

This assessment has the distinct feature of assessing children in four settings within the preschool, either on one-on-one basis or in group settings (Fig. 2). The settings and items of the tool are tabulated in Additional file 3 [see Additional file 3].

#### Step 1.4- The development of the REACh assessment

To ensure uniformity of the assessments, a complete linguistically and culturally adapted tool kit including test items, a stimulus booklet, a story book, response booklet, a demonstration video and an administration manual including detailed instructions on how to conduct the assessment was designed.

The instructions in the administration manual was checked for clarity and accuracy by each member of the research team and was finalized together. Each test item, stimulus book, story lines in the story book, and content in the manual and the record sheets were scrutinized by preschool teachers and the early childhood education officers at training workshops and were re-phrased for clarity during several rounds.

### Phase 2– Testing and training of the preschool teachers

#### Step 2.1- Pre-testing the REACh assessment

For the pre-testing of the revised tool in phase two, 30 children were recruited from two settings; a preschool and a clinic for children with disabilities in the Gampaha District of Sri Lanka (10 per each age group). Each child underwent assessments by two trained RAs at the same time, with one conducting the assessment and the other scoring.

#### Step 2.2- Pilot testing the REACh assessment

The tool was pilot-tested among 25 preschool teachers from the Western Province of Sri Lanka who underwent a single day training programme of 7 h.

#### Step 2.3- Training of preschool teachers to administer the preliminary iteration of the REACh assessment

Five two-day training workshops (10 h each) were held in four provinces of Sri Lanka (Western, Northern, North Central, and Southern provinces). The workshops included didactic sessions and detailed video-based training on tool administration on day 1 followed by hands-on-assessment training of children within a preschool setting in the local area on day 2 under the supervision of the research team. Each of the 160 teachers underwent training with a minimum of three children including one from each age range.

### Phase 3- Assessing psychometric properties of the preliminary iteration of the REACh assessment

#### Step 3.1—Internal consistency

The REACh assessment was administered by 160 preschool teachers on 1809 children (aged 24 – 60 months) from March 2018 to April 2019. These preschools were selected randomly from 24 districts of Sri Lanka using a computer-generated software programme. The selected teachers underwent training to administer the REACh assessment. The teachers collected data from all or randomly selected children attending their respective preschools based on the relevant age groups. Assessments were conducted using the preliminary iteration of the REACh tool. Each child underwent classroom observations over a period of two weeks by the teachers and the assessments in the other three settings were completed on a single day. Internal consistency was measured using the data obtained from these assessments.

#### Step 3.2 – Inter-rater reliability

The REACh tool was administered independently by the preschool teacher and as well as the RA from the research team on different days to 185 preschool children approximately 10% of the 1809 sample mentioned above. The outcomes of the two assessments were compared and the percentage of agreement was calculated. The items completed through daily observations were not included in the analysis as the RAs did not have access to the daily observations of the children over 2 weeks.

#### Step 3.3- Concurrent validity

The preliminary iteration of the REACh tool was administered by RAs at two selected preschools in the Gampaha District and at the multi-disciplinary clinic of the Faculty of Medicine, University of Kelaniya. Fifteen children from the 24–35 month age band and 30 children each from 36–47 and 48–60 month age bands were recruited and assessed. The items that needed to be observed were completed by the preschool teachers while items requiring administration were conducted by RAs for each child.

Due to the lack of availability of a standardized and a validated local assessment tool, a gold standard, clinical developmental assessment was conducted by a developmental pediatrician [14]. This included clinical assessments in cognitive, language, motor and social-emotional and adaptive domains in all the children who were assessed by the RA and the preschool teacher. For each domain, the pediatrician classified the developmental skills of the child as: 1 = no difficulty; 2 = minimal difficulty; 3 = mild difficulty; 4 = moderate difficulty; and 5 = severe difficulty. The results of the pediatrician assessment were classified as “yes” for referral if a child obtained a score of 3 or more for any of the skills assessed. Concurrent validity was assessed comparing this data with the results of the completed REACh assessment in the same sample of children.

### Data analysis

All data obtained in phase 2 were analyzed using the Statistical Package for Social Sciences (V.22) software package.

### Stakeholder contributions

Preschool teachers who were also the primary participants of this study contributed towards the assessment development from the preliminary stages of the study. The proposed assessment was conceptually presented to them prior to designing the assessment tool and their suggestions were incorporated. During the pilot phase further feedback was obtained and included into the final assessment kit used in the preliminary iteration of this study. The instructions within the manual were expanded and the administration time of the assessment was reduced by simplifying the story book activities. Some of the pre-academic skills suggested by the teachers were included in the assessment. Therefore, the study was iterative and open to suggestions by the intended primary users of the assessment. Further involving the ministerial level officials was also important by including Early Childhood Development Officers who will be implementing the use of this assessment.

### Ethics

Ethics clearance was obtained from the Ethics Review Committee of the Faculty of Medicine, University of Kelaniya, Sri Lanka (ERC no. P 131/06/2018). Permission to conduct the assessments was obtained from the preschools and the child development and disability clinic at the Faculty of Medicine, University of Kelaniya. Informed written consent was obtained from parents and preschool teachers. All participants of the pilot study and the main study were informed of the results of the study through a seminar and their participation was recognized by awarding a certificate.

## Results

### Phase 1- Assessment development

#### Review of literature

Eleven assessments were identified for item selection. A summary of the research conducted in LMICs using the identified assessments has been included in Table 1. The Child Health & Development Record which is the national health monitoring booklet issued by the Family Health Bureau of the Ministry of Health used for local developmental screening was also included [15].

**Table 1:** The number of items selected and adapted from each reviewed assessment for each age group

Name of the tool reviewed	24 – 35 months				36 –47 months				48– 60 months			
	**C**	**M**	**L**	**SEA**	**C**	**M**	**L**	**SEA**	**C**	**M**	**L**	**SEA**
Child Health Development Record [15]	-	0	0	-	-	0	0	-	1	1	1	2
Bayley Scales of Infant and Toddler Development III [16, 17]	4	5	4	4	6	3	6	3	-	-	-	-
Modified checklist for Autism [18]	2	0	0	2	0	0	0	3	-	-	-	-
Ages and stages questionnaire [19–21]	-	-	1	2	-	-	1	5	2	0	2	4
Receptive-Expressive Emergent Language Test [22, 23]	-	-	5	-	-	-	4	-	4	0	4	0
Denver II [24, 25]	0	0	0	0	1	0	0	0	1	1	0	1
Early Development Index [26]	0	0	0	0	1	0	0	2	3	0	3	4
Rossetti Infant Toddler Language Scales [27]	-	-	1	-	-	-	0	-	-	-	-	-
Hawaii Early Learning Profile [28]	0	3	0	0	0	4	0	0	2	4	0	0
Strength and Difficulties Questionnaire [29]	-	-	-	0	-	-	-	0	0	0	0	1
Auditory Skills Checklist [30]	1	0	3	1	1	0	3	1	1	0	3	1
**REACh assessment**	**7**	**8**	**14**	**9**	**11**	**7**	**14**	**14**	**13**	**6**	**13**	**13**

[-] Domain not included in the tool

*C* Cognitive, *M* Motor, *L* Language and hearing, *SEA* Social-emotional and Adaptive

#### Content validation by professionals

A total of 542 items representing all age groups and cognitive, social-emotional and adaptive, language and motor domains were selected from all reviewed assessments for cultural adaptation. The number of items selected and the source of the items are tabulated by age group in Table 1.

### Adaptation of REACh assessment to the Sri Lankan context

#### REACh assessment, tool kit and items included

**Fig. 3:**
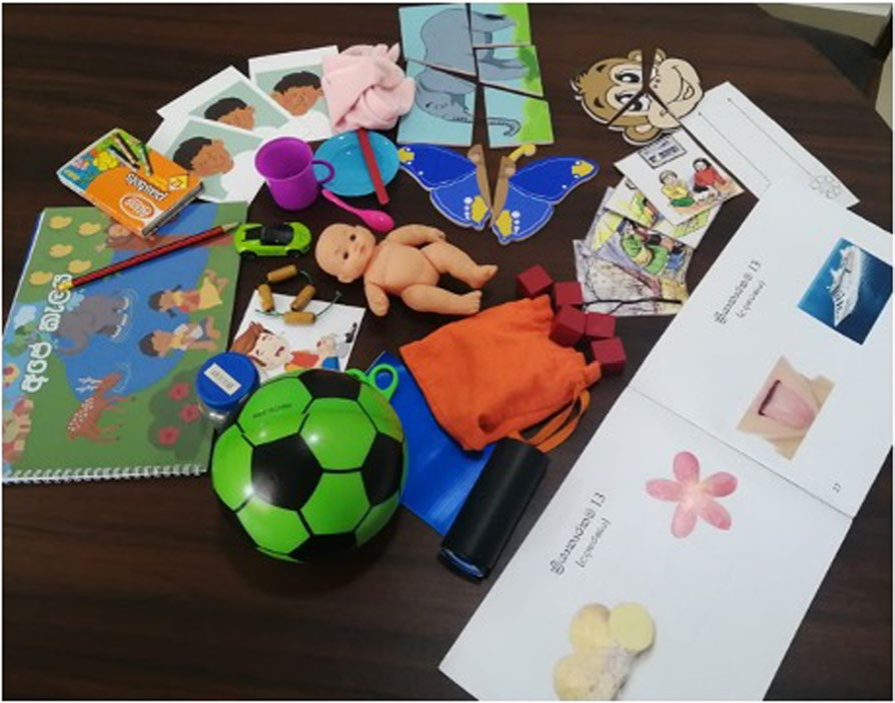
Image of the Preliminary Iteration of the REACh assessment

The assessment together with the material required for administration is referred to as the tool kit. All items contained in the assessment were locally produced and purchased, and matched for size and consistency against the standard international items. The materials used are non-hazardous and safe for children. There is no cultural or ethnic bias in the toys or images used, but the main figures, animals, fruits and food are locally relevant as well as to any other South Asian setting. The entire REACh assessment is contained within a large hand bag for ease of transportation and storage (Fig. 3).

##### Story book

The story book assesses language, cognition and social-emotional skills through story reading.

##### Stimulus booklet

This booklet includes explicit instructions with images and illustrations to assess cognitive and language skills. Some content in the Sinhala, Tamil and English versions are variable to accommodate the differences in the language assessments.

##### Administration manual and response booklet

The administration manual gives explicit instructions on administering and scoring. The response booklet also provides simple instructions on how to elicit an item or expected observations. Each item is scored on a binary system (0 or 1). The manual and the record sheets are colour coded according to the age ranges.

The administration manual, story book and response booklet are in all three languages (Sinhala, Tamil and English).

##### Administration video

An administration training video includes all administered items and demonstrates steps and instructions.

### Phase 2- Testing and training of the preschool teachers

Following the pilot testing, a few test items in the instruction manual were revised in terms of images and instructions, and the teacher training was extended for two days for the training conducted in phase 2. Hundred and sixty teachers from 24 districts of Sri Lanka underwent training during five workshops conducted island-wide.

### Phase 3- Assessing psychometric properties of the preliminary iteration of the REACh assessment

#### Internal consistency

The internal consistency was assessed based on the sample of children assessed by preschool teachers. Data from a total of 1809 children representing all districts in Sri Lanka were analysed.

Table 2 includes the Cronbach’s alpha values of the items belonging to different developmental domains in each age group. The alpha values of the social-emotional and adaptive, cognitive and language domains across the three age groups indicate acceptable reliability of the items. However, the alpha values of the fine motor and gross motor domains across all age groups are lower indicating less reliability in the items.

**Table 2:** Cronbach’s alpha values by age group and domain

Age group of REACH assessment	Domains of Development				
	**Cognitive**	**Language and hearing**	**Fine motor**	**Gross motor**	**Social-emotional and adaptive skills**
24 – 35 month *N* = 489	0.82	0.76	0.50	0.59	0.75
36- 47 month *N* = 591	0.83	0.75	0.65	0.60	0.70
48 – 60 month *N* = 729	0.86	0.76	0.64	0.57	0.71

#### Inter-rater agreement

The inter-rater agreement was assessed comparing the preschool teachers’ assessment and the assessment by the RA for 185 children, representing 10% of the total number of children included in the national sample. As the students’ age increased, the inter-rater agreement improved and the best agreements were observed in the gross motor domain (see Table 3).

**Table 3:** Inter-rater agreement by age band and domain

Age group of REACh Assessment	Range of % of agreement in each domain of the REACh assessment				
	**Cognitive**	**Language and hearing**	**Fine motor**	**Gross motor**	**Social-emotional and adaptive skills**
**24 – 35 months *N* = 33**	42.4—93.9 Mean = 68.38	45.4- 93.9 Mean = 72.98	42.4–93.9 Mean = 72.98	60.6—84.8 Mean = 60.56	45.4 -72.8 Mean = 69.73
**36- 47 months *N* = 66**	65.1–96.8 Mean = 83.31	65.1–95.4 Mean = 84.71	68.1–100 Mean = 94.45	87.7–100 Mean = 85.70	70.8–100 Mean = 74.13
**48 –60 months *N* = 86**	66.3—95.3 Mean = 82.56	66.3–97.6 Mean = 84.99	65.1–89.6 Mean = 75.62	87.2–100 Mean = 93.05	68.6–95.4 Mean = 92.63

#### Concurrent validity

A sample of children representing each age range were assessed for concurrent validity (*n* = 75).

Table 4 gives the most suitable cutoff values for referral of children based on the scores of the 1^st^ iteration of the REACh assessment considering both sensitivity and specificity. The specificity of the assessment for the 36 – 47 month age group was 0.43, lower than the other age groups. The specificity levels of the other age groups are above 0.67.

**Table 4:** ROC curve concurrent validity analysis

Age group of REACh Assessment and number of participants in validity process	Number of items in the REACh assessment	Area under the curve	Positive if greater than or equal to	Sensitivity	Specificity
24 – 35 months *N* = 15	38	0.80	10	0.88	0.43
			15^*^	**0.88**	**0.71**
			20	0.75	0.71
			22	0.63	0.71
36- 47 months *N* = 30	46	0.71	12	0.88	0.29
			13	0.75	0.29
			15	0.75	0.36
			17^*^	**0.75**	**0.43**
			18	0.69	0.43
48 –60 months *N* = 30	45	0.73	13	0.75	0.56
			15	0.75	0.61
			16^*^	**0.75**	**0.67**
			17	0.67	0.67

^*^suggested cut off value based on sensitivity and specificity

## Discussion

The REACh assessment was developed with the aim of providing a standardized and validated preschool development assessment that would enable teachers to identify children aged 24-60 months with developmental impairments within the preschool setting. The assessment designed was reviewed by various stakeholders and was pretested and piloted prior to psychometric assessments. During the preliminary iteration, the assessment showed acceptable reliability for several domains. The inter-rater agreement was wide specially in the younger age groups and was best for the gross motor domain. The sensitivity of the assessment was adequate to screen for developmental impairments in all age groups though specificity for 36–47 month age group was not satisfactory. Therefore, this linguistically and culturally customized assessment tool designed with multiple stakeholder inputs shows favourable preliminary psychometric properties to enable use in the preschool setting following further development. It is important to identify areas that need further improvement during the next iteration.

### Development of the REACh assessment

The development process of this assessment tool internalized the existing National Preschool Standards Framework and the local stakeholder viewpoints. These are essential steps to embrace when developing a culturally appropriate child development assessment tool [16]. These factors possibly contributed towards better compliance of teachers who had the task of collecting data and to participating in this study. The ground level stakeholders contributed towards the precision of the assessment specially, with regard to the content and clarity of instructions and the suitability of images through a focus group discussion held at the Ministry of Women and Child Affairs. Due to the lack of any locally validated assessments, the REACh assessment could not be tested against another validated assessmentl; however, the expert view point was used as the gold standard to validate the assessment as per recommendations in literature [14].

### Psychometric properties

The preliminary iteration of the assessment demonstrated reasonable concurrent validity and internal consistency. Studies show that a Cronbach’s alpha value over 0.7 is acceptable [17].Though, internal consistency of the assessments for all three age bands in the social-emotional and adaptive, cognitive and language domains show acceptable Cronbach's alpha values, the language scales in this analysis did not distinguish between expressive and receptive communication. This is an essential distinction the preschool teacher is required to make for several reasons. Identifying the specific language subset as expressive or receptive will direct the preschool teacher to distinguish between aetiologies while devising definitive interventions approaches. Though they were culturally adapted, the lack of norms and reference levels for the majority of language skills in children of this age group possibly contributed towards this finding. The gross and fine motor domains showed poor internal consistency for all age bands. According to studies, the Cronbach's alpha levels can be improved by increasing the number of items in that particular domain of analysis or by removing items that show poor inter-relatedness or heterogeneous constructs [20]. Hence, such areas need reconsideration during the next reiteration.

The inter-rater reliability between the preschool teachers and the RAs was poor in some age groups indicated by kappa values < 0.6 [21]. Inter-rater reliability depends on the assessor, items and the child [22]. In this study, there were possible variations in all three. The well trained RAs were professionally qualified speech and language therapists and psychologists with extensive knowledge and experience in child development. The preschool teachers in the Sri Lankan education system obtain a minimum of secondary school education with very little exposure to scientific child developmental assessments. A possible child related factor is the unfamiliarity of the child with the RA. The item analysis also showed that the inter-rater reliability improved with the age of children, further indicating child related factors and experience because when children are older they are more compliant with assessments and observations are easier, enabling the assessor to identify the skills easily. The fact that the gross motor items showed the best inter-rater agreement also demonstrates how the ease of items can impact the results. Assessment development is best during multiple iterations as these will consider such factors in each cyclel; hence, it is important to follow such rigorous methodology to improve the reliability of the assessment [53].

The sensitivity and specificity of a developmental screening assessment is recommended to be 70% [23]. The sensitivity of the REACh assessment is satisfactory when considering a total score for each age group. However, the specificity was acceptable only for the 24–35 age range. A higher sensitivity was given more weightage so that fewer children who actually needed further assessments will not be missed; however, when using this strategy, false positives are more likely. The specificity of this assessment is low and needs further improvement.

## Limitations

This study followed scientific steps to design an appropriate, reliable and a validated developmental screening tool for the preschool children in Sri Lanka. However, we acknowledge several limitations in this preliminary iteration of the assessment. There were limitations in the psychometric properties of the tool. The internal consistency for the gross motor domain was low and we could not obtain internal consistency for receptive and expressive language domains separately. Ideally the internal consistency of the assessment must be calculated prior to assessing reliability and validity. In the event the internal consistency is insufficient, the assessment can be further improved prior to studying the reliability and validity of the assessment. However, in this study, internal consistency, inter-rater reliability and concurrent validity were conducted simultaneously. The next cycle of tool development will be conducted following the sequence of tool development.

This indicates the need to improve the items in these domains in the next iteration. In the inter-rater reliability there was poor agreement between teachers and the research team specifically in the younger age groups possibly indicating the need for more robust training in the next round. Further the social emotional and adaptive skills were not included in the inter-rater assessment as it was not possible to observe the students by the RAs over two weeks. It was decided to get a parental check list to be filled at home similar to other assessments presently used globally [25]. Authors comprehend the risks of low specificity in one age group that may result in overwhelming demands from the health system, hence it is important to consider improving the specificity of the overall assessment. There is also a need to develop domain specific cutoffs as the teachers are expected to build the skills in children with the identification of concerns. We also recognized the need to increase the number of children to be included in the concurrent validation in the future iterations. Adding more items and testing the validity on a larger sample is likely to improve the sensitivity and specificity. The authors have taken necessary steps to include all these in the next iteration tested at present.

## Conclusion

The REACh assessment fulfills the need for a culturally adapted developmental screening tool for preschool children in Sri Lanka. It was well accepted by the local stakeholders and was culturally appropriate when tested in all geographical areas of the island. It is a unique assessment looking at four settings within the preschool. As the preliminary iteration this assessment demonstrated acceptable sensitivity as a screening tool for all age groups. However, there is a requirement to improve validity and specificity of the entire assessment and address the internal consistency of some of the domain specific items.

The preliminary iteration of the REACh assessment demonstrated promising results that will enable preschool teachers to screen for children who need early intervention and nurture them for school readiness.

## Supplementary Information

**Additional file 1.** 

**Additional file 2.** 

**Additional file 3.**

## Data Availability

The datasets generated and/ or analyzed during the current study are not publicly available due to the inclusion of personal identifiers, but are available from the corresponding author on reasonable request.

## Abbreviations

LIC: Low Income Country
LMIC: Lower Middle Income Country
NDD: Neuro-Developmental Disorder
NPSF: National Preschool Standards Framework
RA: Research Assistant
REACh: Ragama Early Assessment of Children
SDG: Sustainable Development Goal
