# Supplementary material for: REACh for the preschoolers; a developmental assessment tool for 2–5 year old children in Sri Lanka
Source: BMC Pediatr. 2023 Feb 16;23:80. doi: 10.1186/s12887-023-03895-5 (PMC9933303; doi:10.1186/s12887-023-03895-5)
Supplement: Supplementary file 1 — Additional file 1. [file 12887_2023_3895_MOESM1_ESM.docx]

**Additional file 1**

**(Location : Page 5, Line 125)**

**Key principles and strategies used in the conceptualization of the REACh assessment**

| **Key principles at conceptualization** | |
| --- | --- |
| **Key principles** | **Strategies used to address key principles** |
| 1. The tool will be used by pre-school teachers at national level. | Collaborating with the Ministry of Women and Child Affairs which is the main stakeholder governing pre-schools in Sri Lanka. |
| 1. The tool should be aligned with the national standards set by the Ministry of Women’s and Child Affairs. | Used the national standards set by the same Ministry as the framework for the tool |
| 1. The tool should discriminate children with age appropriate skills from children with a range of mild to severe impairments and disabilities to enable early intervention in the pre-school. | Reviewing multiple tools and selecting culturally relevant items to detect difficulties in all domains of development according to the age appropriate skills |
| 1. The tool should include adequate items from cognitive, language (receptive and expressive), motor (fine and gross) and social emotional and adaptive skills domains. | Included fair distribution of all items and re-confirming the grouping using Cronbach’s alpha. |
| 1. There should be items to assess higher cognitive functions that lay the foundation for more productive adults such as problem-solving skills and literacy training. | Higher cognitive skills such as story reading, comprehension, problem solving were included as assessment items. |
| 1. The items should not be limited to table top tasks but to different settings in the preschool to get a more holistic evaluation of the child. | Items were based on a variety of activities/tasks such as storybook reading, daily observations and outdoor tasks. |
| 1. The tool should contain items that will assess any hearing associated concerns early even prior to an impairment in language proficiency. | The tool included items that assess auditory skills and hearing. |
| 1. The tool should be administered within the classroom setting with the least interference to the classroom activities. | Designed a tool which includes classroom observations and many items to assess the child during play and storytelling which are regular activities within the class room. |
| 1. All items in the tool should be culturally sensitive and specific and applicable to the diversity of the different ethnicities and social strata of Sri Lankan communities. | The tool was available in three languages (Sinhala, Tamil, English) and included culturally sensitive items and illustrations that are applicable to all communities in Sri Lanka. |
| 1. The tool should improve knowledge of pre-school teachers on child development and enhance the early detection of developmental delays/disabilities. | Used didactic teacher training through lectures presentations and hands on experiential training prior to assessing children using the developed tool. |
| 1. The non-specialist pre-school teachers should be able to assess all items in the tool with ease and confidence following adequate training: an user friendly tool. | Incorporated the feedback from the preschool teachers during tool development. Furthermore, the tool was also pilot tested and inter-rater was also assessed. |
