# Supplementary material for: REACh for the preschoolers; a developmental assessment tool for 2–5 year old children in Sri Lanka
Source: BMC Pediatr. 2023 Feb 16;23:80. doi: 10.1186/s12887-023-03895-5 (PMC9933303; doi:10.1186/s12887-023-03895-5)
Supplement: Supplementary file 2 — Additional file 2. [file 12887_2023_3895_MOESM2_ESM.docx]

| **Domains in NPSF** | **Subdomains** | **Specific aspects** | **Domain in REACh Tool** |
| --- | --- | --- | --- |
| Health and physical development | Health | Healthy living practices and personal safety | Social emotional development and adaptive skills |
|  | Motor | Gross motor and fine motor | Motor |
| Social and emotional development | Social | Relationhsip with adults, relationship with peers, prosocial behavior | Social emotional development and adaptive skills |
|  | emotional | self concept, self control , self expression |  |
| Cognitive development | approaches to learning | curiosity, initiative and persisitance, creativity and imagination, problem solving | cognitive development |
|  | logical thinking and mathematics | cause and effect, comaprison and measurement, spatial thinking, number |  |
|  | environmental awareness | Inquiring mind, awareness of the living world, awareness of nonliving world, awareness of the community |  |
| Development of language and early literacy | language | receptive language, expressive language, language comprehension | Language and hearing |
|  | Early literacy | awareness of print, pre reading, prewriting |  |

**Additional file 2**

**Domains included in National Preschool Standards Framework (NPSF) and REACh assessment**

**Location: Page 7 Line 162**
