# Supplementary material for: REACh for the preschoolers; a developmental assessment tool for 2–5 year old children in Sri Lanka
Source: BMC Pediatr. 2023 Feb 16;23:80. doi: 10.1186/s12887-023-03895-5 (PMC9933303; doi:10.1186/s12887-023-03895-5)
Supplement: Supplementary file 3 — Additional file 3. [file 12887_2023_3895_MOESM3_ESM.docx]

| **24- 35 month age group**  **Additional File 3**  **Location : Page 8 , Line 172**  **Setting of administration and items** | | | | |
| --- | --- | --- | --- | --- |
| **Domain** | **Daily observations** | **Classroom Activities** | **Story Book Activities** | **Outdoor Activities** |
| **Cognitive** | Does the child respond to his/her name? | Is the child able to make a tower using 3 blocks? | Is the child able to imitate? |  |
|  |  | Is the child able to solve a 2 piece puzzle? | Is the child able to understand basic verbs? |  |
|  |  | Is the child able to name small parts of the body? | Can the child count to two? |  |
|  |  | Is the child able to engage in pretend play? | Can the child name a colour? |  |
|  |  | Is the child able to draw a horizontal and vertical line? | Is the child able to understand prepositions? |  |
|  |  | is the child able to idenitfy similar soundning words? | Is the child able to understand big and small? |  |
|  |  |  | Is the child able to explain a cause and effect? |  |
|  |  |  | Is the child able to desribe a picture? |  |
|  |  |  | Is the child attentive during the story session? |  |
| **Language and hearing** | Does the child respond to his/her name? | is the child able to selct a favourite toy from a group of toys? | Is the child able to understand basic verbs? |  |
|  | Does the child have ear complications? | Is the child able to name small parts of the body? | Can the child count to two? |  |
|  | Does the child have frequent colds? | is the child able to carryout a two step instruction? | Can the child name a colour? |  |
|  |  | is the child able to idenitfy similar soundning words? | Is the child able to understand prepositions? |  |
|  |  |  | Is the child able to understand big and small? |  |
|  |  |  | Is the child attentive during the story session? |  |
|  | Is the child able to express needs, likes and dislikes? | Is the child able to recite hs/her name? | Is the child able to desribe a picture? |  |
|  |  |  | Does the child use 2 word sentences? |  |
| **Fine**  **Motor** |  | Is the child able to make a tower using 3 blocks? | Is the child able to turn a page? |  |
|  |  | Is the child able to solve a 2 piece puzzle? |  |  |
|  |  | is the child able to open the lid of a bottle? |  |  |
|  |  | Is the child able to draw a horizontal and vertical line? |  |  |
| **Gross**  **Motor** |  |  |  | Is the child able to walk on tip toes? |
|  |  |  |  | Is the child able to jump forawrd? |
|  |  |  |  | Is the child able to kick a ball? |
|  |  |  |  | Is the child able to catch a ball? |
| **Social Emotional** | Is the child able to adjust to the school enviroment when the parent leaves? | Is the child able to recite hs/her name? | Is the child able to explain a cause and effect? |  |
|  | Can the child feed herself/himself finger food? | is the child able to selct a favourite toy from a group of toys? | Is the child able to show a sad face |  |
|  | Is the child able to wash hands independently when requested by the teacher? |  | Is the child able to desribe a picture? |  |
|  | Can the child change from one activity to another? |  | Does the child use 2 word sentences? |  |
|  | Does the child help to clean up after play time on the request of the teacher? |  |  |  |
|  | Is the child able to adjust to changes classroom routine? |  |  |  |
|  | Is the child able to avoid bumping on to walls and objects while walking? |  |  |  |
|  | Does the child interact with other while playing? |  |  |  |
|  | Is the child able to express needs, likes and dislikes? |  |  |  |

| **36-47 month age group** | | | | |
| --- | --- | --- | --- | --- |
| **Domain** | **Daily observations** | **Classroom Activities** | **Story Book Activities** | **Outdoor Activities** |
| **Cognitive** | Does the child respond to his/her name? | Can the child make a bridge using 3 building blocks? | Is the child able to imitate? |  |
|  |  | is the child able to make a 3 piece puzzle? | Is the child able to understand verbs? |  |
|  |  | Is the child able to state the parts of the body according to the function? | Is the child able to count to 3? |  |
|  |  | Is the child able to engage in pretend play? | Is the child able to identify the 3 primary colours? |  |
|  |  | Is th child able to describe in own words what has been scribbled or drawn the child? | Is the child able to identify prepositions? |  |
|  |  | Is the child able to name a circle and square? | Is the child able to understand adjectives? |  |
|  |  | Is the child able to copy a circle? | Is the child able identify cause and effect? |  |
|  |  | Is the child able to discriminate and identify similar sounds? | Can the child imitate emotions? |  |
|  |  | Is the child able to identify the different picture? | Is the child able to sequence a story? |  |
|  |  |  | Is the child attentive listning and understanding during the story session? |  |
| **Language and Hearing** | Does the child respond to his/her name? | Is the child able to state the parts of the body according to the function? | Is the child able to count to 3? |  |
|  | Is the child able to express needs, likes and dislikes? | is the child able to follow 3 part instruction? | Is the child able to identify the 3 primary colours? |  |
|  | Does the child have ear complications? | Is th child able to describe in own words what has been scribbled or drawn the child? | Is the child able to identify prepositions? |  |
|  | Does the child have frequent colds? | Is the child able to name a circle and square? | Is the child able to understand adjectives? |  |
|  |  | Is the child able to discriminate and identify similar sounds? | Is the child able to sequence a story? |  |
|  |  |  | Is the child attentive listning and understanding during the story session? |  |
|  |  | IS the child able to recite his/her name? | Is the child able to understand verbs? |  |
|  |  |  | Does the child speak in 3 word setences? |  |
| **Fine**  **Motor** |  | Can the child make a bridge using 3 building blocks? |  |  |
|  |  | Is the child able to pass 3 beads through a cord? |  |  |
|  |  | Is the child able to cut along a line? |  |  |
|  |  | is the child able to make a 3 piece puzzle? |  |  |
|  |  | Is the child able to copy a circle? |  |  |
| **Gross**  **Motor** |  |  |  | Can the child stand on one leg for 3 seconds? |
|  |  |  |  | Is the child able to jump approxlymately 12 inches in one place? |
|  |  |  |  | Is the child able to walk on a 10ft on a 4 inches wide line? |
|  |  |  |  | Is the child able to throw the ball over head? |
| **Social Emotional** | does the child wet him/herself during school hours? | IS the child able to recite his/her name? | Is the child able identify cause and effect? |  |
|  | Is the child able to adjust to the school enviroment when the parent leaves? | Is the child able to select their favourite type of food from given options? | Can the child imitate emotions? |  |
|  | Is the child able to get the lunch box from the bag independently when requested? | Is th child able to describe in own words what has been scribbled or drawn the child? | Does the child speak in 3 word setences? |  |
|  | Is the child able to wash hands independently when requested by the teacher? |  |  |  |

| **48 – 60 month age group** | | | | |
| --- | --- | --- | --- | --- |
| **Domain** | **Daily observations** | **Classroom Activities** | **Story Book Activities** | **Outdoor Activities** |
| **Cognitive** | Does the child respond to his/her name? | Can the child build a staircase using building blocks? | Is the child able to imitate? |  |
|  |  | is the child able to make a 5 piece puzzle? | Is the child able to name verbs? |  |
|  |  | Is the child able to identify the missing item? | Is the child able to count to 5? |  |
|  |  | Is the child able to state the parts of the body according to the function? | Is the child able to identify colours? |  |
|  |  | Is the child able to engage in pretend play? | Is the child able to identify prepositions? |  |
|  |  | Is the child able to draw a man? | Is the child able to understand adjectives? |  |
|  |  | Is the child able to identify shapes? | Is the child able identify cause and effect? |  |
|  |  | Is the child able to draw shapes? | Is the child able to retell the story? |  |
|  |  | Is the child able to identify similar sounds? | Can the child listen to a story? |  |
|  |  | Is the child able to identify the different picture? |  |  |
| **Language and Hearing** | Does the child respond to his/her name? | Can the child name their favourite type of food? | Is the child able to count to 5? |  |
|  | Does the child have ear complications? | Is the child able to identify the missing item? | Is the child able to identify colours? |  |
|  | Does the child have frequent colds? | Is the child able to state the parts of the body according to the function? | Is the child able to identify prepositions? |  |
|  |  | is the child able to follow 3 part instruction? | Is the child able to understand adjectives? |  |
|  |  | Is the child able to identify shapes? |  |  |
|  |  | Is the child able to identify similar sounds? |  |  |
|  |  | IS the child able to recite his/her name? | Is the child able to name verbs? |  |
|  |  |  | Is the child able to retell the story? |  |
|  |  |  | Does the child speak in 4 word setences? |  |
| **Fine**  **Motor** |  | Can the child build a staircase using building blocks? |  |  |
|  |  | is the child able to make a 5 piece puzzle? |  |  |
|  |  | Is the child able to cut along a circle? |  |  |
|  |  | Is the child able to draw a man? |  |  |
|  |  | Is the child able to draw shapes? |  |  |
| **Gross**  **Motor** |  |  |  | Can the child stand on one legfor 5 seconds? |
|  |  |  |  | Is the child able to hop forward? |
|  |  |  |  | Is the child able to kick a ball? |
|  |  |  |  | Is the child able to bounce and catch a balll? |
| S**ocial**  **Emotional** | does the child wet him/herself during school hours? | IS the child able to recite his/her name? | Is the child able identify cause and effect? |  |
|  | Is the child able to adjust to the school enviroment when the parent leaves? | Can the child name their favourite type of food? | Can the child answer creatively? |  |
|  | Is the child able to pack his/her own bag? |  | Does the child speak in 4 word setences? |  |
|  | Is the child able use the washroom independantly? |  | Can the child listen to a story? |  |
|  | Does the child play aggresively? |  |  |  |
|  | Can the child change from one activity to another? |  |  |  |
|  | Does the child avoid putting familiar toys in the mouth? |  |  |  |
|  | Does the child put away toys? |  |  |  |
|  | Is the child able to adjust to changes? |  |  |  |
|  | Does the child avoid climbing onto high and dangerous places? |  |  |  |
|  | Is the child able to avoid bumping on to walls and objects while walking? |  |  |  |
|  | Does the child share things? |  |  |  |
|  | Is the child able to wait for his or her turn? |  |  |  |
